# Supplementary material for: Liver Function Tests in COVID-19: Assessment of the Actual Prognostic Value
Source: J Clin Med. 2022 Aug 1;11(15):4490. doi: 10.3390/jcm11154490 (PMC9369475; doi:10.3390/jcm11154490)
Supplement: Supplementary file 1 [file jcm-11-04490-s001.zip › jcm-1689996-supplementary.pdf]

Table S1. Model for NLR adjusted for comorbidities and potential risk factors, n=401.

	ICU admission							Mechanical ventilation							Fatal outcome						
	OR	Cl 95%		p-value	p-value for model	NG	LR p-value	OR	Cl 95%		p-value	p-value for model	NG	LR p-value	OR	Cl 95%		p-value	p-value for model	NG	LR p-value
		2.5%	97.5%						2.5%	97.5%						2.5%	97.5%				
NLR	1.033	1.015	1.060	0.001	<0.0001	0.278	<0.0001	1.032	1.014	1.050	0.001	<0.0001	0.280	<0.0001	1.031	1.011	1.060	0.006	<0.0001	0.179	<0.0001
Hypertension	1.141	0.688	1.900	0.609				1.420	0.850	2.390	0.183				1.581	0.944	2.670	0.083			
Diabetes -1	0.045	<0.001	0.850	0.146				0.055	<0.001	0.900	0.143				0.523	0.069	2.580	0.466			
Diabetes -2	1.160	0.604	2.210	0.652				1.186	0.611	2.280	0.610				1.355	0.736	2.470	0.325			
Diabetes -3	1.468	0.536	3.910	0.444				1.650	0.593	4.500	0.328				1.005	0.394	2.450	0.991			
Diabetes - 4	1.518	0.349	6.400	0.566				1.520	0.347	6.490	0.567				2.733	0.673	11.270	0.152			
Asthma	1.281	0.376	4.260	0.683				0.938	0.259	3.130	0.918				0.716	0.154	2.470	0.626			
COPD	<0.001	<0.001	>1000	0.992				<0.001	<0.001	>1000	0.991				1.117	0.263	4.180	0.873			
Dementia	<0.001	<0.001	>1000	0.988				<0.001	<0.001	>1000	0.988				3.509	1.461	8.830	0.006			
Stroke/TIA in patient history	0.533	0.194	1.320	0.193				0.595	0.217	1.480	0.283				0.948	0.427	2.040	0.892			
Chronic kiedney disease	0.316	0.147	0.640	0.002				0.306	0.140	0.630	0.002				0.753	0.407	1.360	0.356			
Active smoker	0.611	0.149	2.130	0.458				0.464	0.096	1.710	0.282				1.022	0.326	2.970	0.969			
Former smoker	0.427	0.107	1.400	0.185				0.327	0.068	1.160	0.110				0.776	0.244	2.250	0.651			
Myocardial infraction in patient history	1.452	0.659	3.180	0.350				1.345	0.594	3.010	0.471				2.496	1.271	4.960	0.008			
Heart failure	0.762	0.351	1.600	0.481				0.544	0.238	1.180	0.132				1.387	0.727	2.630	0.316			
Leukemia	3.416	0.795	15.690	0.097				1.254	0.236	5.500	0.771				5.147	1.264	25.860	0.028			
Solid malignant disease without metastases	0.706	0.242	1.900	0.501				0.597	0.196	1.630	0.332				1.425	0.566	3.530	0.443			
Solid malignant disease with metastases	<0.001	<0.001	>1000	0.993				<0.001	<0.001	>1000	0.993				0.162	0.008	1.000	0.102			
Lymphoma	<0.001	<0.001	>1000	0.992				<0.001	<0.001	>1000	0.992				0.628	0.082	3.100	0.600			
Lenght of hospital stay	1.020	1.006	1.030	0.006				1.019	1.005	1.030	0.008				0.992	0.978	1.010	0.266			

OR: Odds Ratio; 95% CI: Confidence Interval; NG: Nagelkerke pseudo R2; LR: Likelihood Ratio; ICU: intensive care unit; NLR: neutrophil-to-lymphocyte ratio; Diabetes-1:diabetes mellitus type 1. including LADA; Diabates-2: diabetes mellitus type 2 treated with oral medications; Diabetes-3: diabetes mellitus type 2 treated with insulin; Diabtes-4: prediabetes; COPD: chronic obstructive pulmonary disease; TIA: transient ischemic attack

Table S2. Model for TBIL adjusted for comorbidities and potential risk factors, n=401.

	ICU admission							Mechanical ventilation							Fatal outcome						
	**OR**	**Cl 95%**		**p-value**	**p-value for model**	**NG**	**LR p-value**	**OR**	**Cl 95%**		**p-value**	**p-value for model**	**NG**	**LR p-value**	**OR**	**Cl 95%**		**p-value**	**p-value for model**	**NG**	**LR p-value**
		**2.5%**	**97.5%**						**2.5%**	**97.5%**						**2.5%**	**97.5%**				
TBIL	0.965	0.698	1.270	0.805	0.001	0.237	<0.0001	0.985	0.717	1.300	0.919	0.00042	0.240	<0.0001	1.598	1.189	2.250	0.004	<0.0001	0.177	<0.0001
Hypertension	1.177	0.716	1.940	0.522				1.452	0.876	2.430	0.150				1.712	1.021	2.900	0.043			
Diabetes -1	0.238	0.012	1.500	0.197				0.246	0.012	1.570	0.210				0.722	0.132	3.100	0.678			
Diabetes -2	1.084	0.566	2.050	0.805				1.104	0.572	2.110	0.766				1.481	0.804	2.710	0.204			
Diabetes -3	1.206	0.447	3.140	0.704				1.366	0.499	3.630	0.534				0.935	0.368	2.270	0.884			
Diabetes - 4	1.354	0.318	5.610	0.672				1.360	0.317	5.710	0.670				2.832	0.703	11.520	0.135			
Asthma	1.524	0.459	4.970	0.480				1.132	0.320	3.700	0.839				0.970	0.210	3.320	0.965			
COPD	<0.001	<0.001	>1000	0.992				<0.001	<0.001	>1000	0.992				0.810	0.179	3.130	0.768			
Dementia	<0.001	<0.001	>1000	0.988				<0.001	<0.001	>1000	0.988				3.438	1.428	8.670	0.007			
Stroke/TIA in patient history	0.647	0.249	1.550	0.345				0.722	0.277	1.740	0.482				0.998	0.452	2.140	0.996			
Chronic kiedney disease	0.332	0.159	0.650	0.002				0.321	0.151	0.640	0.002				0.787	0.427	1.420	0.433			
Active smoker	0.534	0.131	1.840	0.341				0.397	0.081	1.470	0.198				0.940	0.303	2.710	0.911			
Former smoker	0.410	0.105	1.310	0.157				0.317	0.068	1.090	0.095				0.735	0.232	2.130	0.581			
Myocardial infraction in patient history	1.653	0.768	3.560	0.196				1.544	0.703	3.380	0.275				2.728	1.394	5.420	0.004			
Heart failure	0.835	0.392	1.730	0.632				0.609	0.275	1.290	0.206				1.249	0.656	2.360	0.494			
Leukemia	2.906	0.681	13.090	0.146				1.089	0.202	4.780	0.914				4.898	1.198	24.520	0.033			
Solid malignant disease without metastases	0.772	0.265	2.080	0.617				0.647	0.211	1.780	0.417				1.422	0.548	3.600	0.459			
Solid malignant disease with metastases	<0.001	<0.001	>1000	0.993				<0.001	<0.001	>1000	0.993				0.102	0.005	0.710	0.055			
Lymphoma	<0.001	<0.001	>1000	0.992				<0.001	<0.001	>1000	0.992				0.611	0.077	3.110	0.587			
Lenght of hospital stay	1.020	1.007	1.030	0.004				1.020	1.006	1.030	0.005				0.992	0.978	1.010	0.260			

OR: Odds Ratio; 95% CI: Confidence Interval; NG: Nagelkerke pseudo R2; LR: Likelihood Ratio; ICU: intensive care unit; TBIL: total bilirubin; Diabetes-1:diabetes mellitus type 1. including LADA; Diabates-2: diabetes mellitus type 2 treated with oral medications; Diabetes-3: diabetes mellitus type 2 treated with insulin; Diabtes-4: prediabetes; COPD: chronic obstructive pulmonary disease; TIA: transient ischemic attack

Table S3. Model for ALP adjusted for comorbidities and potential risk factors, n=401.

	ICU admission	Mechanical ventilation	Fatal outcome

	ICU admission							Mechanical ventilation							Fatal outcome						
	**OR**	**Cl 95%**		**p-value**	**p-value for model**	**NG**	**LR p-value**	**OR**	**Cl 95%**		**p-value**	**p-value for model**	**NG**	**LR p-value**	**OR**	**Cl 95%**		**p-value**	**p-value for model**	**NG**	**LR p-value**
		**2.5%**	**97.5%**						**2.5%**	**97.5%**						**2.5%**	**97.5%**				
ALP	0.999	0.996	1.000	0.410	0.002	0.239	<0.0001	0.999	0.996	1.000	0.486	0.001	0.242	<0.0001	1.002	1.000	1.000	0.087	<0.0001	0.156	0.0005
Hypertension	1.178	0.716	1.950	0.520				1.453	0.876	2.430	0.150				1.602	0.961	2.690	0.073			
Diabetes -1	0.266	0.013	1.750	0.240				0.273	0.014	1.810	0.251				0.485	0.085	2.160	0.370			
Diabetes -2	1.086	0.568	2.050	0.801				1.102	0.572	2.100	0.769				1.347	0.733	2.450	0.332			
Diabetes -3	1.202	0.446	3.130	0.708				1.362	0.498	3.620	0.538				0.938	0.371	2.260	0.889			
Diabetes - 4	1.346	0.317	5.570	0.677				1.350	0.315	5.650	0.677				2.648	0.656	10.840	0.163			
Asthma	1.600	0.482	5.220	0.430				1.173	0.331	3.840	0.794				0.823	0.179	2.800	0.773			
COPD	<0.001	<0.001	>1000	0.992				<0.001	<0.001	>1000	0.992				0.938	0.221	3.490	0.926			
Dementia	<0.001	<0.001	>1000	0.988				<0.001	<0.001	>1000	0.988				3.370	1.410	8.420	0.007			
Stroke/TIA in patient history	0.638	0.246	1.530	0.330				0.714	0.274	1.720	0.467				1.060	0.485	2.260	0.881			
Chronic kiedney disease	0.335	0.161	0.660	0.002				0.323	0.152	0.640	0.002				0.752	0.410	1.350	0.347			
Active smoker	0.526	0.129	1.820	0.330				0.391	0.080	1.450	0.191				0.984	0.319	2.820	0.976			
Former smoker	0.410	0.105	1.310	0.157				0.318	0.068	1.090	0.095				0.743	0.238	2.120	0.590			
Myocardial infraction in patient history	1.643	0.763	3.540	0.202				1.530	0.695	3.350	0.286				2.733	1.402	5.410	0.003			
Heart failure	0.833	0.391	1.720	0.627				0.609	0.275	1.290	0.206				1.427	0.756	2.670	0.268			
Leukemia	2.964	0.693	13.370	0.139				1.098	0.203	4.830	0.905				4.397	1.079	22.010	0.046			
Solid malignant disease without metastases	0.815	0.278	2.220	0.696				0.684	0.221	1.900	0.481				1.400	0.548	3.500	0.472			
Solid malignant disease with metastases	<0.001	<0.001	>1000	0.993				<0.001	<0.001	>1000	0.993				0.132	0.007	0.850	0.074			
Lymphoma	<0.001	<0.001	>1000	0.992				<0.001	<0.001	>1000	0.992				0.596	0.080	2.880	0.555			
Lenght of hospital stay	1.021	1.007	1.040	0.003				1.021	1.007	1.040	0.004				0.992	0.978	1.000	0.236			

OR: Odds Ratio; 95% CI: Confidence Interval; NG: Nagelkerke pseudo R2; LR: Likelihood Ratio; ICU: intensive care unit; ALP:alkaline phosphatase; Diabetes-1:diabetes mellitus type 1. including LADA; Diabates-2: diabetes mellitus type 2 treated with oral medications; Diabetes-3: diabetes mellitus type 2 treated with insulin; Diabtes-4: prediabetes; COPD: chronic obstructive pulmonary disease; TIA: transient ischemic attack

Table S4. Model for ALT adjusted for comorbidities and potential risk factors, n=401.

	**ICU admission**	**Mechanical ventilation**	**Fatal outcome**

	OR	CI 95%		p-value	p-value for model	NG	LR p-value	OR	CI 95%		p-value	p-value for model	NG	LR p-value	OR	CI 95%		p-value	p-value for model	NG	LR p-value
		2.5%	97.5%						2.5%	97.5%						2.5%	97.5%				
ALT	1.000	0.998	1.000	0.553	0.0002	0.238	<0.0001	1.000	0.999	1.000	0.464	0.0001	0.242	<0.0001	1.002	1.000	1.000	0.133	<0.0001	0.155	0.0005
Hypertension	1.190	0.725	1.980	0.486				1.480	0.890	2.480	0.133				1.685	1.009	2.840	0.048			
Diabetes -1	0.241	0.012	1.520	0.201				0.249	0.013	1.590	0.215				0.636	0.116	2.740	0.566			
Diabetes -2	1.100	0.575	2.070	0.777				1.110	0.578	2.120	0.747				1.341	0.731	2.440	0.338			
Diabetes -3	1.190	0.441	3.110	0.723				1.340	0.489	3.570	0.560				0.887	0.351	2.130	0.792			
Diabetes - 4	1.280	0.323	5.720	0.654				1.380	0.322	5.800	0.654				2.662	0.658	10.930	0.162			
Asthma	1.440	0.420	4.790	0.547				1.040	0.282	3.490	0.949				0.719	0.144	2.550	0.641			
COPD	<0.001	<0.001	>1000	0.992				<0.001	<0.001	>1000	0.992				0.986	0.233	3.650	0.983			
Dementia	<0.001	<0.001	>1000	0.987				<0.001	<0.001	>1000	0.987				3.053	1.250	7.750	0.016			
Stroke/TIA in patient history	0.660	0.254	1.580	0.368				0.740	0.283	1.780	0.516				1.056	0.482	2.250	0.889			
Chronic kiedney disease	0.336	0.161	0.660	0.002				0.324	0.153	0.650	0.002				0.774	0.423	1.390	0.396			
Active smoker	0.538	0.132	1.850	0.347				0.400	0.082	1.480	0.200				0.935	0.300	2.690	0.903			
Former smoker	0.412	0.106	1.320	0.159				0.321	0.069	1.100	0.098				0.750	0.241	2.140	0.601			
Myocardial infraction in patient history	1.680	0.780	3.620	0.183				1.570	0.714	3.440	0.258				2.711	1.394	5.350	0.004			
Heart failure	0.825	0.388	1.700	0.607				0.604	0.273	1.280	0.198				1.437	0.762	2.690	0.258			
Leukemia	2.950	0.693	13.280	0.139				1.110	0.205	4.860	0.896				4.600	1.128	23.020	0.040			
Solid malignant disease without metastases	0.768	0.265	2.050	0.608				0.649	0.212	1.770	0.418				1.597	0.639	3.930	0.307			
Solid malignant disease with metastases	<0.001	<0.001	>1000	0.993				<0.001	<0.001	>1000	0.993				0.173	0.009	1.030	0.109			
Lymphoma	<0.001	<0.001	>1000	0.992				<0.001	<0.001	>1000	0.992				0.613	0.082	2.960	0.575			
Lenght of hospital stay	1.020	1.010	1.030	0.004				1.020	1.010	1.030	0.005				0.993	0.979	1.010	0.315			

OR: Odds Ratio; 95% CI: Confidence Interval; NG: Nagelkerke pseudo R2; LR: Likelihood Ratio; ICU: intensive care unit; ALT:alanine aminotransferase; Diabetes-1:diabetes mellitus type 1. including LADA; Diabates-2: diabetes mellitus type 2 treated with oral medications; Diabetes-3: diabetes mellitus type 2 treated with insulin; Diabtes-4: prediabetes; COPD: chronic obstructive pulmonary disease; TIA: transient ischemic attack

Table S5. Model for AST adjusted for comorbidities and potential risk factors, n=401.

	ICU admission	Mechanical ventilation	Fatal outcome

	ICU admission							Mechanical ventilation							Fatal outcome						
	**OR**	**Cl 95%**		**p-value**	**p-value for model**	**NG**	**LR p-value**	**OR**	**Cl 95%**		**p-value**	**p-value for model**	**NG**	**LR p-value**	**OR**	**Cl 95%**		**p-value**	**p-value for model**	**NG**	**LR p-value**
		**2.5%**	**97.5%**						**2.5%**	**97.5%**						**2.5%**	**97.5%**				
AST	1.000	0.998	1.000	0.601	0.0003	0.237	<0.0001	1.000	0.999	1.000	0.319	0.0001	0.243	<0.0001	1.004	1.001	1.010	0.008	<0.0001	0.178	<0.0001
Hypertension	1.190	0.722	1.960	0.499				1.480	0.890	2.480	0.133				1.743	1.039	2.960	0.037			
Diabetes -1	0.239	0.012	1.510	0.200				0.248	0.013	1.590	0.213				0.629	0.114	2.720	0.558			
Diabetes -2	1.100	0.574	2.070	0.780				1.110	0.578	2.120	0.747				1.338	0.726	2.440	0.345			
Diabetes -3	1.180	0.434	3.090	0.739				1.300	0.471	3.480	0.604				0.760	0.291	1.870	0.559			
Diabetes - 4	1.380	0.323	5.700	0.656				1.390	0.323	5.820	0.649				2.739	0.670	11.350	0.153			
Asthma	1.480	0.439	4.880	0.511				1.050	0.286	3.510	0.934				0.650	0.112	2.480	0.572			
COPD	<0.001	<0.001	>1000	0.992				<0.001	<0.001	>1000	0.992				1.002	0.236	3.720	0.997			
Dementia	<0.001	<0.001	>1000	0.987				<0.001	<0.001	>1000	0.984				2.976	1.206	7.600	0.019			
Stroke/TIA in patient history	0.649	0.250	1.550	0.348				0.727	0.279	1.750	0.490				1.028	0.465	2.210	0.945			
Chronic kiedney disease	0.342	0.163	0.680	0.003				0.337	0.158	0.680	0.003				0.860	0.466	1.550	0.621			
Active smoker	0.544	0.134	1.880	0.356				0.410	0.084	1.520	0.213				0.951	0.298	2.770	0.929			
Former smoker	0.413	0.106	1.320	0.160				0.322	0.069	1.110	0.099				0.762	0.242	2.200	0.625			
Myocardial infraction in patient history	1.680	0.779	3.610	0.184				1.580	0.718	3.460	0.253				2.805	1.437	5.560	0.003			
Heart failure	0.821	0.386	1.700	0.601				0.597	0.269	1.270	0.190				1.377	0.725	2.590	0.323			
Leukemia	2.970	0.696	13.340	0.138				1.130	0.209	4.950	0.877				5.026	1.225	25.290	0.030			
Solid malignant disease without metastases	0.753	0.260	2.020	0.582				0.627	0.205	1.720	0.383				1.512	0.594	3.780	0.376			
Solid malignant disease with metastases	<0.001	<0.001	>1000	0.993				<0.001	<0.001	>1000	0.993				0.179	0.009	1.080	0.118			
Lymphoma	<0.001	<0.001	>1000	0.992				<0.001	<0.001	>1000	0.992				0.675	0.090	3.290	0.655			
Lenght of hospital stay	1.020	1.010	1.030	0.004				1.020	1.010	1.030	0.005				0.993	0.979	1.010	0.321			

OR: Odds Ratio; 95% CI: Confidence Interval; NG: Nagelkerke pseudo R2; LR: Likelihood Ratio; ICU: intensive care unit; AST:aspartate aminotransferase; Diabetes-1:diabetes mellitus type 1. including LADA; Diabates-2: diabetes mellitus type 2 treated with oral medications; Diabetes-3: diabetes mellitus type 2 treated with insulin; Diabtes-4: prediabetes; COPD: chronic obstructive pulmonary disease; TIA: transient ischemic attack

Table S6. Model for GGT adjusted for comorbidities and potential risk factors, n=401.

	ICU admission	Mechanical ventilation	Fatal outcome

	OR	Cl 95%		p-value	p-value for model	NG	LR p-value	OR	Cl 95%		p-value	p-value for model	NG	LR p-value	OR	Cl 95%		p-value	p-value for model	NG	LR p-value
		2.5%	97.5%						2.5%	97.5%						2.5%	97.5%				
GGT	1.000	0.999	1.000	0.735	0.0003	0.237	<0.0001	1.000	0.999	1.000	0.598	0.0001	0.241	<0.0001	1.001	1.000	1.000	0.220	<0.0001	0.150	0.001
Hypertension	1.182	0.718	1.950	0.512				1.460	0.880	2.440	0.145				1.655	0.994	2.790	0.055			
Diabetes -1	0.236	0.012	1.490	0.195				0.242	0.012	1.550	0.205				0.611	0.111	2.630	0.532			
Diabetes -2	1.090	0.571	2.060	0.791				1.105	0.574	2.100	0.763				1.325	0.723	2.400	0.357			
Diabetes -3	1.203	0.446	3.130	0.707				1.350	0.494	3.590	0.549				0.893	0.354	2.150	0.804			
Diabetes - 4	1.365	0.320	5.670	0.664				1.367	0.317	5.750	0.665				2.613	0.649	10.680	0.168			
Asthma	1.530	0.462	4.970	0.474				1.129	0.320	3.670	0.842				0.845	0.184	2.870	0.804			
COPD	<0.001	<0.001	>1000	0.992				<0.001	<0.001	>1000	0.992				0.949	0.223	3.520	0.939			
Dementia	<0.001	<0.001	>1000	0.988				<0.001	<0.001	>1000	0.988				3.393	1.418	8.490	0.007			
Stroke/TIA in patient history	0.656	0.252	1.570	0.362				0.738	0.282	1.780	0.513				1.054	0.480	2.250	0.894			
Chronic kiedney disease	0.335	0.161	0.660	0.002				0.323	0.152	0.640	0.002				0.765	0.418	1.370	0.376			
Active smoker	0.532	0.131	1.840	0.338				0.394	0.081	1.460	0.193				0.929	0.303	2.650	0.893			
Former smoker	0.405	0.103	1.300	0.152				0.312	0.067	1.070	0.091				0.727	0.234	2.070	0.562			
Myocardial infraction in patient history	1.674	0.778	3.610	0.186				1.568	0.713	3.440	0.259				2.694	1.384	5.320	0.004			
Heart failure	0.827	0.389	1.710	0.612				0.607	0.275	1.280	0.202				1.434	0.761	2.680	0.261			
Leukemia	2.935	0.688	13.210	0.142				1.097	0.203	4.820	0.906				4.514	1.109	22.560	0.042			
Solid malignant disease without metastases	0.757	0.261	2.030	0.589				0.636	0.208	1.740	0.396				1.535	0.614	3.780	0.351			
Solid malignant disease with metastases	<0.001	<0.001	>1000	0.993				<0.001	<0.001	>1000	0.993				0.173	0.009	1.040	0.110			
Lymphoma	<0.001	<0.001	>1000	0.992				<0.001	<0.001	>1000	0.992				0.597	0.079	2.910	0.557			
Lenght of hospital stay	1.020	1.007	1.030	0.004				1.020	1.006	1.030	0.005				0.993	0.979	1.010	0.289			

OR: Odds Ratio; 95% CI: Confidence Interval; NG: Nagelkerke pseudo R2; LR: Likelihood Ratio; ICU: intensive care unit; GGT: gamma-glutamyltransferase; Diabetes-1:diabetes mellitus type 1. including LADA; Diabates-2: diabetes mellitus type 2 treated with oral medications; Diabetes-3: diabetes mellitus type 2 treated with insulin; Diabtes-4: prediabetes; COPD: chronic obstructive pulmonary disease; TIA: transient ischemic attack

Table S7. Model for albumin adjusted for comorbidities and potential risk factors, n=401.

	ICU admission	Mechanical ventilation	Fatal outcome

	OR	Cl 95%		p-value	p-value for model	NG	LR p-value	OR	Cl 95%		p-value	p-value for model	NG	LR p-value	OR	Cl 95%		p-value	p-value for model	NG	LR p-value
		2.5%	97.5%						2.5%	97.5%						2.5%	97.5%				
albumin	0.281	0.172	0.450	<0.0001	0.0001	0.324	<0.0001	0.247	0.148	0.400	<0.0001	<0.0001	0.341	<0.0001	0.233	0.143	0.370	<0.0001	<0.0001	0.274	<0.0001
Hypertension	1.113	0.659	1.890	0.689				1.435	0.838	2.480	0.191				1.551	0.908	2.680	0.111			
Diabetes -1	0.202	0.010	1.280	0.149				0.202	0.010	1.300	0.152				0.601	0.116	2.470	0.502			
Diabetes -2	1.077	0.546	2.100	0.829				1.081	0.542	2.130	0.824				1.290	0.673	2.450	0.437			
Diabetes -3	1.251	0.449	3.370	0.661				1.413	0.496	3.940	0.509				0.944	0.356	2.370	0.905			
Diabetes - 4	1.701	0.375	7.520	0.479				1.746	0.377	7.920	0.464				3.519	0.827	15.210	0.084			
Asthma	1.649	0.467	5.720	0.425				1.171	0.307	4.120	0.808				0.901	0.189	3.230	0.882			
COPD	<0.001	<0.001	>1000	0.991				<0.001	<0.001	>1000	0.991				0.707	0.147	2.870	0.639			
Dementia	<0.001	<0.001	>1000	0.987				<0.001	<0.001	>1000	0.987				1.946	0.748	5.200	0.175			
Stroke/TIA in patient history	0.646	0.242	1.600	0.359				0.701	0.258	1.770	0.465				1.188	0.532	2.600	0.668			
Chronic kiedney disease	0.264	0.124	0.530	0.000				0.244	0.112	0.500	0.000				0.575	0.300	1.070	0.087			
Active smoker	0.666	0.153	2.510	0.562				0.484	0.095	1.950	0.333				1.541	0.445	4.960	0.478			
Former smoker	0.424	0.103	1.440	0.194				0.310	0.062	1.160	0.107				0.779	0.229	2.430	0.676			
Myocardial infraction in patient history	1.565	0.706	3.450	0.266				1.436	0.632	3.230	0.382				2.670	1.326	5.470	0.006			
Heart failure	0.879	0.402	1.870	0.740				0.621	0.270	1.360	0.246				1.769	0.903	3.450	0.094			
Leukemia	4.540	0.989	22.500	0.052				1.625	0.281	7.880	0.558				8.126	1.762	47.320	0.010			
Solid malignant disease without metastases	0.589	0.193	1.660	0.330				0.474	0.143	1.400	0.194				1.255	0.478	3.240	0.638			
Solid malignant disease with metastases	<0.001	<0.001	0.993	0.993				<0.001	<0.001	>1000	0.993				0.099	0.005	0.660	0.044			
Lymphoma	<0.001	<0.001	>1000	0.992				<0.001	<0.001	>1000	0.992				0.978	0.122	5.390	0.981			
Lenght of hospital stay	1.013	0.998	1.030	0.089				1.012	0.997	1.030	0.118				0.983	0.968	1.000	0.030			

OR: Odds Ratio; 95% CI: Confidence Interval; NG: Nagelkerke pseudo R2; LR: Likelihood Ratio; ICU: intensive care unit; Diabetes-1:diabetes mellitus type 1. including LADA; Diabates-2: diabetes mellitus type 2 treated with oral medications; Diabetes-3: diabetes mellitus type 2 treated with insulin; Diabtes-4: prediabetes; COPD: chronic obstructive pulmonary disease; TIA: transient ischemic attack

Table S8. Model for ALP and albumin adjusted for comorbidities and potential risk factors, n=401.

	ICU admission							Mechanical ventilation							Fatal outcome						
	OR	Cl 95%		p-value	p-value for model	NG	LR p-value	OR	Cl 95%		p-value	p-value for model	NG	LR p-value	OR	Cl 95%		p-value	p-value for model	NG	LR p-value

		2.5%	97.5%						2.5%	97.5%						2.5%	97.5%				
ALP	0.998	0.995	1.000	0.199	<0.0001	0.330	<0.0001	0.998	0.995	1.000	0.227	<0.0001	0.346	<0.0001	1.001	0.999	1.000	0.255	<0.0001	0.278	<0.0001
albumin	0.270	0.165	0.430	<0.001				0.238	0.142	0.390	<0.001				0.239	0.147	0.380	<0.001			
Hyperte+A183+A189	1.115	0.658	1.890	0.687				1.438	0.839	2.490	0.189				1.534	0.896	2.650	0.121			
Diabetes -1	0.244	0.012	1.640	0.212				0.249	0.012	1.700	0.220				0.500	0.091	2.150	0.376			
Diabetes -2	1.065	0.538	2.090	0.855				1.070	0.535	2.120	0.847				1.306	0.680	2.480	0.417			
Diabetes -3	1.233	0.441	3.330	0.682				1.398	0.489	3.910	0.523				0.964	0.364	2.420	0.939			
Diabetes - 4	1.671	0.369	7.380	0.493				1.716	0.371	7.780	0.478				3.559	0.835	15.400	0.081			
Asthma	1.767	0.494	6.220	0.369				1.236	0.320	4.420	0.747				0.880	0.185	3.150	0.855			
COPD	<0.001	<0.001	>1000	0.991				<0.001	<0.001	>1000	0.991				0.707	0.149	2.860	0.638			
Dementia	<0.001	<0.001	>1000	0.987				<0.001	<0.001	>1000	0.987				1.992	0.765	5.320	0.160			
Stroke/TIA in patient history	0.631	0.236	1.560	0.335				0.686	0.252	1.730	0.438				1.222	0.548	2.680	0.618			
Chronic kiedney disease	0.262	0.122	0.530	<0.001				0.241	0.110	0.500	<0.001				0.575	0.300	1.070	0.088			
Active smoker	0.645	0.148	2.430	0.530				0.469	0.092	1.880	0.312				1.565	0.450	5.050	0.463			
Former smoker	0.428	0.104	1.450	0.198				0.315	0.063	1.170	0.111				0.777	0.227	2.440	0.674			
Myocardial infraction in patient history	1.531	0.688	3.390	0.293				1.396	0.612	3.150	0.422				2.753	1.363	5.650	0.005			
Heart failure	0.889	0.405	1.900	0.765				0.626	0.271	1.380	0.255				1.747	0.892	3.410	0.102			
Leukemia	4.642	1.016	22.970	0.048				1.675	0.291	8.070	0.531				8.072	1.734	47.240	0.011			
Solid malignant disease without metastases	0.664	0.213	1.920	0.460				0.539	0.160	1.630	0.290				1.168	0.436	3.060	0.753			
Solid malignant disease with metastases	<0.001	<0.001	0.993	0.993				<0.001	<0.001	>1000	0.993				0.076	0.003	0.560	0.032			
Lymphoma	<0.001	<0.001	>1000	0.992				<0.001	<0.001	>1000	0.992				1.004	0.126	5.530	0.997			
Lenght of hospital stay	1.014	0.999	1.030	0.074				1.013	0.998	1.030	0.101				0.983	0.967	1.000	0.027			

OR: Odds Ratio; 95% CI: Confidence Interval; NG: Nagelkerke pseudo R2; LR: Likelihood Ratio; ICU: intensive care unit; ALP:alkaline phosphatase; Diabetes-1:diabetes mellitus type 1. including LADA; Diabates-2: diabetes mellitus type 2 treated with oral medications; Diabetes-3: diabetes mellitus type 2 treated with insulin; Diabtes-4: prediabetes; COPD: chronic obstructive pulmonary disease; TIA: transient ischemic attack

Table S9. Model for TBIL and albumin adjusted for comorbidities and potential risk factors, n=401.

	ICU admission							Mechanical ventilation							Fatal outcome						
	OR	Cl 95%		p-value	p-value for model	NG	LR p-value	OR	Cl 95%		p-value	p-value for model	NG	LR p-value	OR	Cl 95%		p-value	p-value for model	NG	LR p-value
		2.5%	97.5%						2.5%	97.5%						2.5%	97.5%				

TBIL	0.930	0.669	1.240	0.627	0.0001	0.325	<0.0001	0.941	0.678	1.260	0.686	<0.0001	0.341	<0.0001	1.705	1.229	2.470	0.003	0.0001	0.304	<0.0001
albumin	0.279	0.170	0.440	<0.001				0.246	0.147	0.400	<0.001				0.228	0.139	0.360	<0.001			
Hypertension	1.115	0.660	1.890	0.685				1.438	0.839	2.480	0.189				1.670	0.966	2.920	0.069			
Diabetes -1	0.201	0.010	1.280	0.149				0.202	0.010	1.300	0.152				0.666	0.128	2.780	0.593			
Diabetes -2	1.059	0.535	2.080	0.867				1.065	0.533	2.110	0.856				1.437	0.745	2.750	0.275			
Diabetes -3	1.240	0.444	3.350	0.674				1.404	0.491	3.920	0.518				0.955	0.361	2.400	0.924			
Diabetes - 4	1.679	0.370	7.430	0.490				1.728	0.373	7.850	0.473				3.849	0.911	16.390	0.062			
Asthma	1.613	0.455	5.610	0.447				1.149	0.300	4.060	0.831				1.032	0.215	3.750	0.964			
COPD	<0.001	<0.001	>1000	0.991				<0.001	<0.001	>1000	0.991				0.554	0.110	2.370	0.443			
Dementia	<0.001	<0.001	>1000	0.987				<0.001	<0.001	>1000	0.987				2.024	0.768	5.490	0.156			
Stroke/TIA in patient history	0.643	0.241	1.590	0.355				0.698	0.257	1.760	0.460				1.191	0.532	2.610	0.665			
Chronic kiedney disease	0.260	0.121	0.530	<0.001				0.240	0.109	0.500	<0.001				0.607	0.315	1.140	0.126			
Active smoker	0.654	0.149	2.480	0.546				0.475	0.092	1.920	0.322				1.504	0.435	4.840	0.503			
Former smoker	0.425	0.103	1.440	0.195				0.311	0.062	1.160	0.108				0.767	0.218	2.470	0.665			
Myocardial infraction in patient history	1.553	0.700	3.430	0.275				1.426	0.627	3.210	0.391				2.785	1.373	5.750	0.005			
Heart failure	0.890	0.406	1.900	0.766				0.626	0.272	1.380	0.255				1.509	0.767	2.950	0.230			
Leukemia	4.483	0.976	22.270	0.054				1.612	0.280	7.820	0.564				9.132	1.976	52.840	0.007			
Solid malignant disease without metastases	0.610	0.198	1.740	0.368				0.491	0.147	1.460	0.219				1.158	0.418	3.130	0.773			
Solid malignant disease with metastases	<0.001	<0.001	0.993	0.993				<0.001	<0.001	>1000	0.993				0.048	0.002	0.390	0.015			
Lymphoma	<0.001	<0.001	>1000	0.992				<0.001	<0.001	>1000	0.992				1.047	0.124	6.140	0.962			
Lenght of hospital stay	1.013	0.998	1.030	0.089				1.012	0.997	1.030	0.118				0.983	0.968	1.000	0.028			

OR: Odds Ratio; 95% CI: Confidence Interval; NG: Nagelkerke pseudo R2; LR: Likelihood Ratio; ICU: intensive care unit; TBIL: total bilirubin; Diabetes-1:diabetes mellitus type 1. including LADA; Diabates-2: diabetes mellitus type 2 treated with oral medications; Diabetes-3: diabetes mellitus type 2 treated with insulin; Diabtes-4: prediabetes; COPD: chronic obstructive pulmonary disease; TIA: transient ischemic attack

Table S10. Model for ALT and albumin adjusted for comorbidities and potential risk factors, n=401.

	**ICU admission**							**Mechanical ventilation**							**Fatal outcome**						
	**OR**	**Cl 95%**		**p-value**	**p-value for model**	**NG**	**LR p-value**	**OR**	**Cl 95%**		**p-value**	**p-value for model**	**NG**	**LR p-value**	**OR**	**Cl 95%**		**p-value**	**p-value for model**	**NG**	**LR p-value**
		**2.5%**	**97.5%**						**2.5%**	**97.5%**						**2.5%**	**97.5%**				
albumin	0.279	0.171	0.440	<0.0001	0.0001	0.326	<0.0001	0.245	0.147	0.400	<0.0001	<0.0001	0.343	<0.0001	0.227	0.139	0.360	<0.0001	<0.0001	0.285	<0.0001

ALT	1.000	0.998	1.000	0.480	1.000	0.999	1.000	0.361	1.002	1.000	1.000	0.086
Hypertension	1.140	0.670	1.930	0.637	1.470	0.857	2.560	0.163	1.623	0.944	2.820	0.082
Diabetes -1	0.205	0.011	1.300	0.153	0.207	0.011	1.330	0.158	0.605	0.116	2.500	0.509
Diabetes -2	1.090	0.550	2.120	0.811	1.090	0.547	2.160	0.801	1.322	0.688	2.520	0.397
Diabetes -3	1.230	0.440	3.320	0.688	1.380	0.484	3.850	0.538	0.914	0.345	2.300	0.852
Diabetes - 4	1.720	0.380	7.640	0.467	1.780	0.384	8.090	0.450	3.673	0.857	15.990	0.075
Asthma	1.500	0.411	5.360	0.526	1.030	0.259	3.750	0.962	0.747	0.149	2.770	0.687
COPD	<0.001	<0.001	>1000	0.991	<0.001	<0.001	>1000	0.991	0.731	0.153	2.970	0.673
Dementia	<0.001	<0.001	>1000	0.986	<0.001	<0.001	>1000	0.985	1.767	0.656	4.850	0.261
Stroke/TIA in patient history	0.661	0.247	1.630	0.385	0.722	0.266	1.820	0.504	1.247	0.557	2.740	0.584
Chronic kiedney disease	0.269	0.126	0.540	0.000	0.249	0.114	0.510	0.000	0.593	0.309	1.110	0.107
Active smoker	0.671	0.154	2.530	0.569	0.489	0.096	1.970	0.341	1.545	0.435	5.030	0.482
Former smoker	0.430	0.104	1.460	0.201	0.316	0.063	1.180	0.113	0.794	0.231	2.490	0.701
Myocardial infraction in patient history	1.590	0.715	3.510	0.253	1.460	0.642	3.290	0.360	2.781	1.376	5.710	0.005
Heart failure	0.871	0.398	1.850	0.724	0.614	0.267	1.350	0.235	1.761	0.899	3.440	0.097
Leukemia	4.590	0.999	22.770	0.050	1.650	0.285	8.020	0.546	8.544	1.831	50.380	0.009
Solid malignant disease without metastases	0.595	0.195	1.680	0.339	0.479	0.145	1.420	0.200	1.288	0.490	3.340	0.602
Solid malignant disease with metastases	<0.001	<0.001	>1000	0.993	<0.001	<0.001	>1000	0.993	0.089	0.004	0.600	0.034
Lymphoma	<0.001	<0.001	>1000	0.992	<0.001	<0.001	>1000	0.992	1.076	0.134	5.980	0.937
Lenght of hospital stay	1.010	0.998	1.030	0.089	1.010	0.997	1.030	0.117	0.984	0.968	1.000	0.037

OR: Odds Ratio; 95% CI: Confidence Interval; NG: Nagelkerke pseudo R2; LR: Likelihood Ratio; ICU: intensive care unit; ALT:alanine aminotransferase; Diabetes-1:diabetes mellitus type 1. including LADA; Diabates-2: diabetes mellitus type 2 treated with oral medications; Diabetes-3: diabetes mellitus type 2 treated with insulin; Diabtes-4: prediabetes; COPD: chronic obstructive pulmonary disease; TIA: transient ischemic attack

Table S11. Model for AST and albumin adjusted for comorbidities and potential risk factors, n=401.

	**ICU admission**							**Mechanical ventilation**							**Fatal outcome**						
	**OR**	**Cl 95%**		**p-value**	**p-value for model**	**NG**	**LR p-value**	**OR**	**Cl 95%**		**p-value**	**p-value for model**	**NG**	**LR p-value**	**OR**	**Cl 95%**		**p-value**	**p-value for model**	**NG**	**LR p-value**
		**2.5%**	**97.5%**						**2.5%**	**97.5%**						**2.5%**	**97.5%**				
albumin	0.281	0.172	0.450	<0.0001	<0.0001	0.324	<0.0001	0.248	0.149	0.400	<0.0001	<0.0001	0.342	<0.0001	0.233	0.142	0.370	<0.0001	<0.0001	0.300	<0.0001
AST	1.000	0.998	1.000	0.790				1.000	0.999	1.000	0.429				1.003	1.001	1.010	0.009			

Hypertension	1.120	0.661	1.900	0.675	1.460	0.850	2.530	0.173	1.666	0.966	2.910	0.069
Diabetes -1	0.203	0.010	1.280	0.151	0.205	0.011	1.320	0.156	0.605	0.116	2.500	0.508
Diabetes -2	1.080	0.547	2.110	0.825	1.090	0.545	2.150	0.811	1.330	0.689	2.540	0.390
Diabetes -3	1.240	0.441	3.350	0.680	1.360	0.476	3.810	0.556	0.804	0.294	2.060	0.657
Diabetes - 4	1.710	0.376	7.550	0.475	1.770	0.382	8.050	0.453	3.729	0.863	16.360	0.074
Asthma	1.610	0.451	5.650	0.450	1.090	0.281	3.900	0.894	0.688	0.125	2.660	0.620
COPD	<0.001	<0.001	>1000	0.991	<0.001	<0.001	>1000	0.991	0.731	0.152	2.990	0.674
Dementia	<0.001	<0.001	>1000	0.987	<0.001	<0.001	>1000	0.985	1.742	0.639	4.820	0.278
Stroke/TIA in patient history	0.647	0.242	1.600	0.360	0.703	0.259	1.770	0.468	1.218	0.539	2.690	0.628
Chronic kiedney disease	0.268	0.125	0.550	<0.001	0.254	0.116	0.530	<0.001	0.650	0.337	1.220	0.190
Active smoker	0.672	0.154	2.530	0.571	0.498	0.097	2.010	0.354	1.587	0.443	5.180	0.457
Former smoker	0.425	0.103	1.440	0.196	0.312	0.062	1.170	0.110	0.810	0.232	2.580	0.729
Myocardial infraction in patient history	1.570	0.709	3.480	0.261	1.460	0.643	3.290	0.360	2.836	1.402	5.840	0.004
Heart failure	0.874	0.399	1.860	0.731	0.611	0.265	1.340	0.231	1.697	0.861	3.330	0.124
Leukemia	4.570	0.994	22.660	0.051	1.660	0.287	8.060	0.541	9.052	1.927	53.920	0.008
Solid malignant disease without metastases	0.586	0.191	1.660	0.325	0.463	0.139	1.380	0.182	1.233	0.459	3.250	0.672
Solid malignant disease with metastases	<0.001	<0.001	>1000	0.993	<0.001	<0.001	>1000	0.993	0.095	0.005	0.640	0.041
Lymphoma	<0.001	<0.001	>1000	0.992	<0.001	<0.001	>1000	0.992	1.144	0.143	6.380	0.885
Lenght of hospital stay	1.010	0.998	1.030	0.089	1.010	0.997	1.030	0.118	0.984	0.968	1.000	0.040

OR: Odds Ratio; 95% CI: Confidence Interval; NG: Nagelkerke pseudo R2; LR: Likelihood Ratio; ICU: intensive care unit; AST:aspartate aminotransferase; Diabetes-1:diabetes mellitus type 1. including LADA; Diabates-2: diabetes mellitus type 2 treated with oral medications; Diabetes-3: diabetes mellitus type 2 treated with insulin; Diabtes-4: prediabetes; COPD: chronic obstructive pulmonary disease; TIA: transient ischemic attack

Table S12. Model for GGT and TBIL adjusted for comorbidities and potential risk factors, n=401.

	ICU admission							Mechanical ventilation							Fatal outcome						
	OR	Cl 95%		p-value	p-value for model	NG	LR p-value	OR	Cl 95%		p-value	p-value for model	NG	LR p-value	OR	Cl 95%		p-value	p-value for model	NG	LR p-value
		2.5%	97.5%						2.5%	97.5%						2.5%	97.5%				
GGT	1.001	0.999	1.000	0.467	0.002	0.238	<0.0001	1.001	0.999	1.000	0.392	0.001	0.243	<0.0001	0.999	0.997	1.000	0.151	<0.0001	0.184	<0.0001
TBIL	0.868	0.553	1.280	0.500				0.867	0.550	1.290	0.503				1.969	1.303	3.080	0.002			
Hypertension	1.191	0.724	1.970	0.493				1.474	0.887	2.470	0.137				1.700	1.013	2.880	0.046			

Diabetes -1	0.229	0.012	1.450	0.186				0.235	0.012	1.500	0.196				0.818	0.150	3.540	0.799			
Diabetes -2	1.053	0.548	2.000	0.876				1.067	0.551	2.050	0.845				1.558	0.839	2.870	0.157			
Diabetes -3	1.173	0.434	3.060	0.746				1.316	0.479	3.510	0.586				0.987	0.387	2.400	0.977			
Diabetes - 4	1.333	0.312	5.550	0.689				1.338	0.310	5.640	0.688				2.899	0.715	11.870	0.128			
Asthma	1.444	0.431	4.740	0.540				1.068	0.300	3.510	0.915				1.030	0.221	3.580	0.965			
COPD	<0.001	<0.001	>1000	0.992				<0.001	<0.001	>1000	0.992				0.743	0.159	2.930	0.682			
Dementia	<0.001	<0.001	>1000	0.988				<0.001	<0.001	>1000	0.988				3.329	1.374	8.450	0.009			
Stroke/TIA in patient history	0.675	0.258	1.620	0.396				0.758	0.289	1.840	0.552				0.934	0.420	2.020	0.865			
Chronic kiedney disease	0.330	0.158	0.650	0.002				0.318	0.149	0.640	0.002				0.790	0.429	1.430	0.442			
Active smoker	0.509	0.124	1.770	0.308				0.376	0.077	1.400	0.174				0.988	0.316	2.870	0.983			
Former smoker	0.396	0.101	1.270	0.144				0.306	0.065	1.060	0.086				0.753	0.236	2.200	0.615			
Myocardial infraction in patient history	1.680	0.780	3.620	0.183				1.574	0.715	3.450	0.255				2.671	1.358	5.330	0.005			
Heart failure	0.852	0.399	1.770	0.671				0.624	0.281	1.330	0.231				1.186	0.618	2.250	0.605			
Leukemia	2.885	0.676	13.010	0.149				1.081	0.201	4.740	0.921				5.019	1.224	25.180	0.030			
Solid malignant disease without metastases	0.788	0.270	2.140	0.647				0.663	0.215	1.830	0.446				1.387	0.530	3.540	0.495			
Solid malignant disease with metastases	<0.001	<0.001	>1000	0.993				<0.001	<0.001	>1000	0.993				0.082	0.003	0.640	0.049			
Lymphoma	<0.001	<0.001	>1000	0.992				<0.001	<0.001	>1000	0.992				0.578	0.071	2.990	0.551			
Lenght of hospital stay	1.021	1.007	1.040	0.004				1.020	1.007	1.030	0.004				0.992	0.978	1.010	0.237			

OR: Odds Ratio; 95% CI: Confidence Interval; NG: Nagelkerke pseudo R2; LR: Likelihood Ratio; ICU: intensive care unit; TBIL: total bilirubin; GGT: gamma-glutamyltransferase; Diabetes-1:diabetes mellitus type 1. including LADA; Diabates-2: diabetes mellitus type 2 treated with oral medications; Diabetes-3: diabetes mellitus type 2 treated with insulin; Diabtes-4: prediabetes; COPD: chronic obstructive pulmonary disease; TIA: transient ischemic attack

Table S13. Model for GGT and ALP adjusted for comorbidities and potential risk factors, n=401.

	ICU admission							Mechanical ventilation							Fatal outcome						
	OR	Cl 95%		p-value	p-value for model	NG	LR p-value	OR	Cl 95%		p-value	p-value for model	NG	LR p-value	OR	Cl 95%		p-value	p-value for model	NG	LR p-value
		2.5%	97.5%						2.5%	97.5%						2.5%	97.5%				
GGT	1.001	0.999	1.000	0.336	0.001	0.242	<0.0001	1.001	0.999	1.000	0.282	0.0004	0.246	<0.0001	1.000	0.999	1.000	0.706	<0.0001	0.156	0.001
ALP	0.998	0.994	1.000	0.253				0.998	0.995	1.000	0.271				1.002	0.999	1.000	0.178			
Hypertension	1.196	0.726	1.980	0.484				1.475	0.888	2.470	0.136				1.616	0.967	2.730	0.069			
Diabetes -1	0.271	0.014	1.780	0.247				0.282	0.014	1.870	0.263				0.494	0.086	2.200	0.383			

Diabetes -2	1.072	0.561	2.030	0.832				1.088	0.565	2.070	0.799				1.344	0.732	2.440	0.335			
Diabetes -3	1.168	0.432	3.040	0.753				1.310	0.477	3.490	0.591				0.928	0.367	2.240	0.871			
Diabetes - 4	1.338	0.313	5.560	0.685				1.343	0.311	5.650	0.684				2.651	0.657	10.850	0.162			
Asthma	1.603	0.483	5.210	0.428				1.182	0.335	3.850	0.783				0.821	0.179	2.790	0.770			
COPD	<0.001	<0.001	>1000	0.992				<0.001	<0.001	>1000	0.992				0.941	0.221	3.500	0.929			
Dementia	<0.001	<0.001	>1000	0.988				<0.001	<0.001	>1000	0.988				3.398	1.420	8.490	0.007			
Stroke/TIA in patient history	0.664	0.254	1.600	0.376				0.746	0.285	1.810	0.529				1.068	0.487	2.280	0.867			
Chronic kiedney disease	0.339	0.163	0.670	0.003				0.327	0.154	0.650	0.002				0.756	0.412	1.360	0.355			
Active smoker	0.499	0.122	1.730	0.293				0.371	0.076	1.380	0.167				0.970	0.314	2.790	0.955			
Former smoker	0.393	0.100	1.260	0.140				0.305	0.065	1.050	0.085				0.738	0.236	2.110	0.582			
Myocardial infraction in patient history	1.686	0.781	3.650	0.182				1.571	0.712	3.450	0.259				2.744	1.407	5.430	0.003			
Heart failure	0.833	0.392	1.720	0.626				0.612	0.277	1.290	0.210				1.424	0.755	2.670	0.271			
Leukemia	3.032	0.706	13.710	0.132				1.119	0.205	4.950	0.886				4.423	1.085	22.150	0.045			
Solid malignant disease without metastases	0.842	0.286	2.310	0.744				0.710	0.229	1.980	0.528				1.413	0.552	3.530	0.461			
Solid malignant disease with metastases	<0.001	<0.001	>1000	0.993				<0.001	<0.001	>1000	0.993				0.134	0.007	0.860	0.075			
Lymphoma	<0.001	<0.001	>1000	0.992				<0.001	<0.001	>1000	0.992				0.604	0.081	2.930	0.565			
Lenght of hospital stay	1.022	1.008	1.040	0.003				1.021	1.007	1.040	0.003				0.992	0.978	1.010	0.244			

OR: Odds Ratio; 95% CI: Confidence Interval; NG: Nagelkerke pseudo R2; LR: Likelihood Ratio; ICU: intensive care unit; GGT: gamma-glutamyltransferase; ALP:alkaline phosphatase; Diabetes-1:diabetes mellitus type 1. including LADA; Diabates-2: diabetes mellitus type 2 treated with oral medications; Diabetes-3: diabetes mellitus type 2 treated with insulin; Diabtes-4: prediabetes; COPD: chronic obstructive pulmonary disease; TIA: transient ischemic attack

Table S14. Model for GGT and ALT adjusted for comorbidities and potential risk factors, n=401.

	ICU admission							Mechanical ventilation							Fatal outcome						
	OR	Cl 95%		p-value	p-value for model	NG	LR p-value	OR	Cl 95%		p-value	p-value for model	NG	LR p-value	OR	Cl 95%		p-value	p-value for model	NG	LR p-value
		2.5%	97.5%						2.5%	97.5%						2.5%	97.5%				
GGT	1.000	0.999	1.000	0.824	0.0003	0.238	<0.0001	1.000	0.999	1.000	0.697	<0.0001	0.242	<0.0001	1.001	0.999	1.000	0.340	<0.0001	0.158	0.001
ALT	1.000	0.998	1.000	0.590				1.000	0.998	1.000	0.519				1.001	1.000	1.000	0.185			
Hypertension	1.200	0.726	1.980	0.482				1.480	0.892	2.490	0.131				1.702	1.018	2.880	0.044			
Diabetes -1	0.239	0.012	1.510	0.199				0.246	0.013	1.570	0.211				0.614	0.111	2.660	0.537			
Diabetes -2	1.100	0.574	2.070	0.781				1.110	0.577	2.110	0.752				1.338	0.729	2.430	0.342			

Diabetes -3	1.190	0.439	3.100	0.728				1.330	0.485	3.540	0.571				0.875	0.346	2.100	0.769			
Diabetes - 4	1.380	0.323	5.720	0.654				1.380	0.321	5.820	0.654				2.674	0.662	10.960	0.159			
Asthma	1.440	0.420	4.780	0.547				1.040	0.283	3.490	0.945				0.717	0.144	2.550	0.638			
COPD	<0.001	<0.001	>1000	0.992				<0.001	<0.001	>1000	0.992				0.988	0.232	3.670	0.986			
Dementia	<0.001	<0.001	>1000	0.987				<0.001	<0.001	>1000	0.987				3.133	1.282	7.960	0.013			
Stroke/TIA in patient history	0.665	0.255	1.600	0.378				0.750	0.287	1.810	0.536				1.081	0.492	2.310	0.843			
Chronic kiedney disease	0.337	0.162	0.660	0.002				0.325	0.153	0.650	0.002				0.778	0.425	1.400	0.407			
Active smoker	0.535	0.131	1.840	0.342				0.396	0.081	1.470	0.196				0.911	0.292	2.630	0.866			
Former smoker	0.409	0.105	1.310	0.157				0.317	0.068	1.090	0.094				0.736	0.236	2.100	0.578			
Myocardial infraction in patient history	1.690	0.783	3.640	0.179				1.580	0.719	3.470	0.249				2.758	1.416	5.450	0.003			
Heart failure	0.824	0.388	1.700	0.606				0.604	0.273	1.280	0.198				1.430	0.759	2.670	0.264			
Leukemia	2.960	0.694	13.310	0.139				1.110	0.206	4.870	0.894				4.636	1.136	23.220	0.039			
Solid malignant disease without metastases	0.764	0.264	2.050	0.602				0.643	0.210	1.760	0.408				1.575	0.629	3.880	0.323			
Solid malignant disease with metastases	<0.001	<0.001	>1000	0.993				<0.001	<0.001	>1000	0.993				0.166	0.009	0.990	0.101			
Lymphoma	<0.001	<0.001	>1000	0.992				<0.001	<0.001	>1000	0.992				0.633	0.084	3.080	0.602			
Lenght of hospital stay	1.020	1.010	1.030	0.004				1.020	1.010	1.030	0.005				0.993	0.979	1.010	0.319			

OR: Odds Ratio; 95% CI: Confidence Interval; NG: Nagelkerke pseudo R2; LR: Likelihood Ratio; ICU: intensive care unit; GGT: gamma-glutamyltransferase; ALT:alanine aminotransferase; Diabetes-1:diabetes mellitus type 1. including LADA; Diabates-2: diabetes mellitus type 2 treated with oral medications; Diabetes-3: diabetes mellitus type 2 treated with insulin; Diabtes-4: prediabetes; COPD: chronic obstructive pulmonary disease; TIA: transient ischemic attack

Table S15. Model for GGT and AST adjusted for comorbidities and potential risk factors, n=401.

	ICU admission							Mechanical ventilation							Fatal outcome						
	OR	Cl 95%		p-value	p-value for model	NG	LR p-value	OR	Cl 95%		p-value	p-value for model	NG	LR p-value	OR	Cl 95%		p-value	p-value for model	NG	LR p-value
		2.5%	97.5%						2.5%	97.5%						2.5%	97.5%				
GGT	1.000	0.999	1.000	0.853	0.0003	0.238	<0.0001	1.000	0.999	1.000	0.821	0.0001	0.243	<0.0001	1.000	0.999	1.000	0.763	<0.0001	0.179	0.0001
AST	1.000	0.998	1.000	0.658				1.000	0.999	1.000	0.378				1.003	1.001	1.010	0.014			
Hypertension	1.190	0.722	1.970	0.496				1.480	0.891	2.480	0.132				1.750	1.043	2.970	0.036			
Diabetes -1	0.238	0.012	1.500	0.198				0.246	0.013	1.570	0.211				0.622	0.112	2.700	0.550			
Diabetes -2	1.090	0.573	2.070	0.783				1.110	0.577	2.120	0.750				1.337	0.726	2.440	0.345			
Diabetes -3	1.180	0.434	3.080	0.740				1.300	0.471	3.470	0.606				0.760	0.292	1.870	0.560			

Diabetes - 4	1.370	0.322	5.710	0.657	1.390	0.323	5.830	0.649	2.740	0.671	11.350	0.153
Asthma	1.490	0.440	4.880	0.511	1.050	0.288	3.510	0.932	0.650	0.113	2.470	0.570
COPD	<0.001	<0.001	>1000	0.992	<0.001	<0.001	>1000	0.992	1.003	0.236	3.730	0.996
Dementia	<0.001	<0.001	>1000	0.987	<0.001	<0.001	>1000	0.985	3.000	1.214	7.670	0.018
Stroke/TIA in patient history	0.654	0.251	1.570	0.358	0.734	0.281	1.770	0.505	1.037	0.468	2.230	0.928
Chronic kiedney disease	0.341	0.163	0.680	0.003	0.336	0.158	0.670	0.003	0.859	0.466	1.550	0.618
Active smoker	0.541	0.133	1.870	0.351	0.407	0.084	1.510	0.210	0.941	0.294	2.750	0.914
Former smoker	0.410	0.105	1.310	0.157	0.319	0.068	1.100	0.097	0.758	0.240	2.190	0.618
Myocardial infraction in patient history	1.680	0.781	3.630	0.182	1.590	0.720	3.480	0.248	2.817	1.443	5.590	0.003
Heart failure	0.822	0.386	1.700	0.601	0.598	0.270	1.270	0.191	1.375	0.725	2.590	0.325
Leukemia	2.970	0.696	13.350	0.138	1.130	0.209	4.950	0.877	5.022	1.224	25.280	0.031
Solid malignant disease without metastases	0.751	0.259	2.010	0.579	0.625	0.203	1.710	0.379	1.514	0.594	3.790	0.375
Solid malignant disease with metastases	<0.001	<0.001	>1000	0.993	<0.001	<0.001	>1000	0.993	0.177	0.009	1.070	0.115
Lymphoma	<0.001	<0.001	>1000	0.992	<0.001	<0.001	>1000	0.992	0.681	0.090	3.320	0.662
Lenght of hospital stay	1.020	1.010	1.030	0.004	1.020	1.010	1.030	0.005	0.993	0.979	1.010	0.322

OR: Odds Ratio; 95% CI: Confidence Interval; NG: Nagelkerke pseudo R2; LR: Likelihood Ratio; ICU: intensive care unit; GGT: gamma-glutamyltransferase; AST:aspartate aminotransferase; Diabetes-1:diabetes mellitus type 1. including LADA; Diabates-2: diabetes mellitus type 2 treated with oral medications; Diabetes-3: diabetes mellitus type 2 treated with insulin; Diabtes-4: prediabetes; COPD: chronic obstructive pulmonary disease; TIA: transient ischemic attack

Table S16. Model for GGT and albumin adjusted for comorbidities and potential risk factors, n=401.

	**ICU admission**							**Mechanical ventilation**							**Fatal outcome**						
	**OR**	**Cl 95%**		**p-value**	**p-value for model**	**NG**	**LR p-value**	**OR**	**Cl 95%**		**p-value**	**p-value for model**	**NG**	**LR p-value**	**OR**	**Cl 95%**		**p-value**	**p-value for model**	**NG**	**LR p-value**
		**2.5%**	**97.5%**						**2.5%**	**97.5%**						**2.5%**	**97.5%**				
GGT	1.000	0.999	1.000	0.864	0.0001	0.324	<0.0001	1.000	6.796	1.000	0.757	<0.0001	0.341	<0.0001	1.001	1.000	1.000	0.227	<0.0001	0.278	<0.0001
albumin	0.281	0.172	0.450	<0.0001				0.248	0.999	0.400	<0.0001				0.233	0.143	0.370	<0.0001			
Hypertension	1.115	0.660	1.890	0.684				1.438	0.840	2.480	0.188				1.580	0.922	2.740	0.098			
Diabetes -1	0.201	0.010	1.270	0.148				0.201	0.010	1.290	0.151				0.578	0.111	2.380	0.470			
Diabetes -2	1.077	0.546	2.100	0.829				1.081	0.542	2.130	0.824				1.301	0.678	2.470	0.423			
Diabetes -3	1.248	0.448	3.370	0.663				1.407	0.494	3.920	0.514				0.930	0.352	2.330	0.880			
Diabetes - 4	1.704	0.375	7.540	0.477				1.753	0.378	7.960	0.461				3.576	0.837	15.530	0.081			

Asthma	1.646	0.466	5.700	0.427				1.169	0.307	4.110	0.809				0.903	0.190	3.230	0.884			
COPD	<0.001	<0.001	>1000	0.991				<0.001	<0.001	>1000	0.991				0.709	0.148	2.880	0.642			
Dementia	<0.001	<0.001	>1000	0.987				<0.001	<0.001	>1000	0.987				2.010	0.771	5.380	0.155			
Stroke/TIA in patient history	0.651	0.243	1.610	0.368				0.710	0.261	1.800	0.482				1.232	0.550	2.700	0.605			
Chronic kiedney disease	0.265	0.124	0.540	0.000				0.245	0.112	0.510	0.000				0.585	0.305	1.090	0.098			
Active smoker	0.665	0.153	2.500	0.560				0.484	0.094	1.940	0.333				1.503	0.435	4.820	0.503			
Former smoker	0.423	0.102	1.430	0.192				0.309	0.062	1.150	0.106				0.774	0.226	2.420	0.670			
Myocardial infraction in patient history	1.572	0.708	3.480	0.262				1.448	0.636	3.260	0.372				2.744	1.359	5.630	0.005			
Heart failure	0.879	0.402	1.870	0.740				0.622	0.271	1.360	0.246				1.748	0.894	3.410	0.101			
Leukemia	4.546	0.989	22.540	0.052				1.624	0.281	7.890	0.558				8.265	1.776	48.570	0.010			
Solid malignant disease without metastases	0.587	0.192	1.660	0.327				0.470	0.142	1.390	0.190				1.234	0.468	3.210	0.665			
Solid malignant disease with metastases	<0.001	<0.001	0.993	0.993				<0.001	<0.001	>1000	0.993				0.090	0.004	0.620	0.038			
Lymphoma	<0.001	<0.001	>1000	0.992				<0.001	<0.001	>1000	0.992				1.028	0.128	5.730	0.976			
Lenght of hospital stay	1.013	0.998	1.030	0.089				1.012	0.997	1.030	0.116				0.983	0.968	1.000	0.031			

OR: Odds Ratio; 95% CI: Confidence Interval; NG: Nagelkerke pseudo R2; LR: Likelihood Ratio; ICU: intensive care unit; GGT: gamma-glutamyltransferase; Diabetes-1:diabetes mellitus type 1. including LADA; Diabates-2: diabetes mellitus type 2 treated with oral medications; Diabetes-3: diabetes mellitus type 2 treated with insulin; Diabtes-4: prediabetes; COPD: chronic obstructive pulmonary disease; TIA: transient ischemic attack

Table S17. Model for ALT and AST adjusted for comorbidities and potential risk factors, n=401.

	**ICU admission**							**Mechanical ventilation**							**Fatal outcome**						
	**OR**	**Cl 95%**		**p-value**	**p-value for model**	**NG**	**LR p-value**	**OR**	**Cl 95%**		**p-value**	**p-value for model**	**NG**	**LR p-value**	**OR**	**Cl 95%**		**p-value**	**p-value for model**	**NG**	**LR p-value**
		**2.5%**	**97.5%**						**2.5%**	**97.5%**						**2.5%**	**97.5%**				
ALT	1.000	0.996	1.000	0.770	0.0003	0.238	<0.0001	0.999	0.995	1.000	0.824	0.0001	0.243	<0.0001	0.993	0.986	1.000	0.026	<0.0001	0.196	<0.0001
AST	1.000	0.996	1.000	0.969				1.000	0.998	1.010	0.470				1.009	1.004	1.020	0.002			
Hypertension	1.190	0.725	1.980	0.487				1.470	0.885	2.470	0.139				1.676	0.994	2.860	0.055			
Diabetes -1	0.240	0.012	1.520	0.201				0.247	0.013	1.580	0.212				0.617	0.111	2.660	0.541			
Diabetes -2	1.100	0.575	2.070	0.777				1.110	0.577	2.120	0.749				1.318	0.711	2.420	0.375			
Diabetes -3	1.190	0.437	3.120	0.728				1.290	0.468	3.460	0.611				0.669	0.248	1.680	0.406			
Diabetes - 4	1.380	0.323	5.720	0.654				1.390	0.323	5.820	0.650				2.648	0.642	11.070	0.170			
Asthma	1.440	0.420	4.820	0.547				1.080	0.289	3.640	0.908				0.862	0.164	3.190	0.839			

COPD	<0.001	<0.001	>1000	0.992	<0.001	<0.001	>1000	0.992	0.943	0.221	3.530	0.933
Dementia	<0.001	<0.001	>1000	0.987	<0.001	<0.001	>1000	0.984	2.791	1.120	7.170	0.029
Stroke/TIA in patient history	0.659	0.253	1.580	0.368	0.718	0.274	1.740	0.477	0.929	0.412	2.030	0.856
Chronic kiedney disease	0.337	0.160	0.670	0.003	0.340	0.159	0.690	0.004	0.926	0.500	1.680	0.804
Active smoker	0.539	0.132	1.860	0.349	0.414	0.085	1.530	0.218	1.000	0.314	2.920	1.000
Former smoker	0.413	0.106	1.320	0.160	0.321	0.069	1.110	0.099	0.757	0.235	2.230	0.624
Myocardial infraction in patient history	1.680	0.780	3.620	0.183	1.580	0.717	3.450	0.254	2.735	1.394	5.450	0.004
Heart failure	0.824	0.387	1.700	0.607	0.596	0.268	1.260	0.188	1.367	0.714	2.590	0.341
Leukemia	2.960	0.693	13.300	0.139	1.130	0.210	4.970	0.873	5.382	1.309	27.150	0.024
Solid malignant disease without metastases	0.766	0.263	2.060	0.607	0.618	0.199	1.710	0.372	1.442	0.553	3.660	0.442
Solid malignant disease with metastases	<0.001	<0.001	>1000	0.993	<0.001	<0.001	0.000	0.993	0.204	0.010	1.320	0.159
Lymphoma	<0.001	<0.001	>1000	0.992	<0.001	<0.001	0.000	0.992	0.675	0.089	3.300	0.655
Lenght of hospital stay	1.020	1.010	1.030	0.004	1.020	1.010	1.030	0.005	0.993	0.978	1.010	0.295

OR: Odds Ratio; 95% CI: Confidence Interval; NG: Nagelkerke pseudo R2; LR: Likelihood Ratio; ICU: intensive care unit; ALT:alanine aminotransferase; AST:aspartate aminotransferase; Diabetes-1:diabetes mellitus type 1. including LADA; Diabates-2: diabetes mellitus type 2 treated with oral medications; Diabetes-3: diabetes mellitus type 2 treated with insulin; Diabtes-4: prediabetes; COPD: chronic obstructive pulmonary disease; TIA: transient ischemic attack

Table S18. Model for ALP and AST adjusted for comorbidities and potential risk factors, n=401.

	ICU admission							Mechanical ventilation							Fatal outcome						
	**OR**	**Cl 95%**		**p-value**	**p-value for model**	**NG**	**LR p-value**	**OR**	**Cl 95%**		**p-value**	**p-value for model**	**NG**	**LR p-value**	**OR**	**Cl 95%**		**p-value**	**p-value for model**	**NG**	**LR p-value**
		**2.5%**	**97.5%**						**2.5%**	**97.5%**						**2.5%**	**97.5%**				
ALP	0.998	0.995	1.000	0.269	0.001	0.242	<0.0001	0.998	0.995	1.000	0.243	0.0003	0.248	<0.0001	1.001	0.999	1.000	0.463	<0.0001	0.180	0.0001
AST	1.000	0.999	1.000	0.336				1.000	0.999	1.000	0.163				1.003	1.001	1.010	0.020			
Hypertension	1.200	0.729	1.990	0.471				1.500	0.901	2.520	0.121				1.729	1.029	2.940	0.040			
Diabetes -1	0.280	0.014	1.850	0.259				0.296	0.015	1.990	0.284				0.558	0.098	2.500	0.471			
Diabetes -2	1.090	0.569	2.060	0.798				1.100	0.572	2.110	0.766				1.344	0.729	2.450	0.338			
Diabetes -3	1.140	0.415	2.990	0.796				1.250	0.450	3.380	0.657				0.781	0.299	1.920	0.600			
Diabetes - 4	1.360	0.321	5.630	0.665				1.380	0.321	5.750	0.658				2.748	0.672	11.390	0.151			
Asthma	1.510	0.443	4.970	0.499				1.060	0.287	3.570	0.924				0.648	0.112	2.470	0.568			
COPD	<0.001	<0.001	>1000	0.992				<0.001	<0.001	>1000	0.992				0.997	0.234	3.710	0.997			

Dementia	<0.001	<0.001	>1000	0.984				<0.001	<0.001	>1000	0.979				3.009	1.220	7.680	0.018			
Stroke/TIA in patient history	0.637	0.245	1.520	0.328				0.712	0.273	1.720	0.464				1.045	0.473	2.250	0.912			
Chronic kiedney disease	0.352	0.168	0.700	0.004				0.348	0.164	0.700	0.004				0.848	0.459	1.530	0.592			
Active smoker	0.535	0.131	1.840	0.342				0.403	0.083	1.490	0.205				0.958	0.300	2.800	0.940			
Former smoker	0.417	0.106	1.330	0.165				0.326	0.070	1.120	0.103				0.761	0.241	2.200	0.624			
Myocardial infraction in patient history	1.670	0.773	3.600	0.190				1.560	0.709	3.440	0.264				2.834	1.451	5.620	0.003			
Heart failure	0.821	0.384	1.700	0.602				0.595	0.267	1.270	0.190				1.371	0.723	2.580	0.329			
Leukemia	3.070	0.718	13.850	0.127				1.160	0.214	5.120	0.847				4.928	1.201	24.810	0.033			
Solid malignant disease without metastases	0.835	0.284	2.280	0.730				0.708	0.229	1.970	0.523				1.473	0.571	3.710	0.412			
Solid malignant disease with metastases	<0.001	<0.001	>1000	0.993				<0.001	<0.001	>1000	0.993				0.154	0.008	0.980	0.096			
Lymphoma	<0.001	<0.001	>1000	0.992				<0.001	<0.001	>1000	0.992				0.681	0.091	3.320	0.662			
Lenght of hospital stay	1.020	1.010	1.040	0.003				1.020	1.010	1.040	0.004				0.993	0.978	1.010	0.295			

OR: Odds Ratio; 95% CI: Confidence Interval; NG: Nagelkerke pseudo R2; LR: Likelihood Ratio; ICU: intensive care unit; ALP:alkaline phosphatase; AST:aspartate aminotransferase; Diabetes-1:diabetes mellitus type 1. including LADA; Diabates-2: diabetes mellitus type 2 treated with oral medications; Diabetes-3: diabetes mellitus type 2 treated with insulin; Diabtes-4: prediabetes; COPD: chronic obstructive pulmonary disease; TIA: transient ischemic attack

Table S19. Model for TBIL and AST adjusted for comorbidities and potential risk factors, n=401.

	**ICU admission**							**Mechanical ventilation**							**Fatal outcome**						
	**OR**	**Cl 95%**		**p-value**	**p-value for model**	**NG**	**LR p-value**	**OR**	**Cl 95%**		**p-value**	**p-value for model**	**NG**	**LR p-value**	**OR**	**Cl 95%**		**p-value**	**p-value for model**	**NG**	**LR p-value**
		**2.5%**	**97.5%**						**2.5%**	**97.5%**						**2.5%**	**97.5%**				
TBIL	0.927	0.661	1.250	0.629	0.001	0.238	<0.0001	0.919	0.657	1.240	0.592	0.0003	0.244	<0.0001	1.471	1.068	2.090	0.023	<0.0001	0.195	<0.0001
AST	1.000	0.998	1.000	0.508				1.000	0.999	1.000	0.270				1.003	1.000	1.010	0.038			
Hypertension	1.190	0.724	1.970	0.490				1.490	0.895	2.490	0.128				1.808	1.070	3.090	0.028			
Diabetes -1	0.239	0.012	1.510	0.199				0.248	0.013	1.580	0.213				0.698	0.127	3.020	0.649			
Diabetes -2	1.080	0.563	2.040	0.818				1.090	0.566	2.090	0.788				1.453	0.785	2.670	0.230			
Diabetes -3	1.160	0.424	3.040	0.768				1.280	0.459	3.430	0.632				0.807	0.309	1.990	0.649			
Diabetes - 4	1.360	0.320	5.640	0.667				1.370	0.320	5.750	0.660				2.886	0.709	11.860	0.131			
Asthma	1.430	0.418	4.760	0.553				1.010	0.270	3.420	0.985				0.752	0.139	2.790	0.700			
COPD	<0.001	<0.001	>1000	0.992				<0.001	<0.001	>1000	0.992				0.883	0.196	3.400	0.861			
Dementia	<0.001	<0.001	>1000	0.986				<0.001	<0.001	>1000	0.982				3.057	1.237	7.840	0.017			

Stroke/TIA in patient history	0.650	0.250	1.550	0.349				0.727	0.279	1.750	0.490				1.007	0.453	2.170	0.987			
Chronic kiedney disease	0.341	0.163	0.680	0.003				0.336	0.158	0.670	0.003				0.858	0.463	1.560	0.619			
Active smoker	0.540	0.133	1.860	0.350				0.406	0.083	1.500	0.208				0.921	0.287	2.710	0.885			
Former smoker	0.415	0.106	1.330	0.163				0.324	0.069	1.110	0.101				0.748	0.234	2.190	0.607			
Myocardial infraction in patient history	1.670	0.775	3.590	0.189				1.570	0.714	3.440	0.257				2.836	1.446	5.650	0.003			
Heart failure	0.833	0.390	1.730	0.629				0.605	0.272	1.290	0.203				1.233	0.645	2.330	0.522			
Leukemia	2.950	0.692	13.250	0.141				1.120	0.209	4.920	0.881				5.236	1.275	26.330	0.026			
Solid malignant disease without metastases	0.774	0.266	2.090	0.622				0.649	0.211	1.790	0.420				1.483	0.569	3.770	0.409			
Solid malignant disease with metastases	<0.001	<0.001	>1000	0.993				<0.001	<0.001	>1000	0.993				0.109	0.005	0.760	0.062			
Lymphoma	<0.001	<0.001	>1000	0.992				<0.001	<0.001	>1000	0.992				0.686	0.088	3.470	0.676			
Lenght of hospital stay	1.020	1.010	1.030	0.004				1.020	1.010	1.030	0.005				0.993	0.978	1.010	0.296			

OR: Odds Ratio; 95% CI: Confidence Interval; NG: Nagelkerke pseudo R2; LR: Likelihood Ratio; ICU: intensive care unit; TBIL: total bilirubin; AST:aspartate aminotransferase; Diabetes-1:diabetes mellitus type 1. including LADA; Diabates-2: diabetes mellitus type 2 treated with oral medications; Diabetes-3: diabetes mellitus type 2 treated with insulin; Diabtes-4: prediabetes; COPD: chronic obstructive pulmonary disease; TIA: transient ischemic attack

Table S20. Model for ALT and ALP adjusted for comorbidities and potential risk factors, n=401.

	**ICU admission**							**Mechanical ventilation**							**Fatal outcome**						
	**OR**	**Cl 95%**		**p-value**	**p-value for model**	**NG**	**LR p-value**	**OR**	**Cl 95%**		**p-value**	**p-value for model**	**NG**	**LR p-value**	**OR**	**Cl 95%**		**p-value**	**p-value for model**	**NG**	**LR p-value**
		**2.5%**	**97.5%**						**2.5%**	**97.5%**						**2.5%**	**97.5%**				
ALT	1.000	0.999	1.000	0.370	0.001	0.241	<0.0001	1.000	0.999	1.000	0.319	0.0004	0.245	<0.0001	1.001	1.000	1.000	0.251	<0.0001	0.162	0.0004
ALP	0.999	0.995	1.000	0.318				0.999	0.995	1.000	0.364				1.001	1.000	1.000	0.156			
Hypertension	1.210	0.731	2.000	0.465				1.490	0.897	2.500	0.126				1.648	0.985	2.790	0.059			
Diabetes -1	0.278	0.014	1.830	0.257				0.288	0.014	1.920	0.272				0.510	0.090	2.270	0.403			
Diabetes -2	1.090	0.571	2.060	0.790				1.110	0.575	2.110	0.757				1.356	0.738	2.470	0.321			
Diabetes -3	1.170	0.431	3.060	0.750				1.320	0.480	3.530	0.580				0.916	0.362	2.210	0.848			
Diabetes - 4	1.370	0.321	5.650	0.663				1.370	0.320	5.740	0.661				2.697	0.667	11.060	0.156			
Asthma	1.460	0.424	4.840	0.537				1.050	0.285	3.520	0.938				0.710	0.142	2.530	0.630			
COPD	<0.001	<0.001	>1000	0.992				<0.001	<0.001	>1000	0.992				0.973	0.229	3.620	0.968			
Dementia	<0.001	<0.001	>1000	0.986				<0.001	<0.001	>1000	0.985				3.127	1.282	7.920	0.013			
Stroke/TIA in patient	0.654	0.251	1.570	0.359				0.734	0.281	1.770	0.506				1.083	0.494	2.310	0.839			

history																					
Chronic kiedney disease	0.339	0.163	0.670	0.003				0.327	0.154	0.650	0.002				0.767	0.418	1.380	0.382			
Active smoker	0.525	0.129	1.810	0.327				0.391	0.080	1.450	0.190				0.959	0.307	2.770	0.940			
Former smoker	0.415	0.106	1.320	0.162				0.324	0.069	1.110	0.100				0.749	0.240	2.140	0.602			
Myocardial infraction in patient history	1.670	0.773	3.600	0.190				1.560	0.706	3.410	0.269				2.780	1.426	5.500	0.003			
Heart failure	0.830	0.389	1.720	0.620				0.606	0.273	1.290	0.203				1.425	0.756	2.670	0.270			
Leukemia	3.030	0.708	13.660	0.132				1.130	0.207	4.950	0.881				4.516	1.106	22.640	0.043			
Solid malignant disease without metastases	0.841	0.286	2.300	0.742				0.709	0.229	1.980	0.525				1.450	0.567	3.620	0.428			
Solid malignant disease with metastases	<0.001	<0.001	>1000	0.993				<0.001	<0.001	>1000	0.993				0.133	0.007	0.840	0.073			
Lymphoma	<0.001	<0.001	>1000	0.992				<0.001	<0.001	>1000	0.992				0.629	0.084	3.040	0.596			
Lenght of hospital stay	1.020	1.010	1.040	0.003				1.020	1.010	1.040	0.004				0.992	0.978	1.010	0.269			

OR: Odds Ratio; 95% CI: Confidence Interval; NG: Nagelkerke pseudo R2; LR: Likelihood Ratio; ICU: intensive care unit; ALP:alkaline phosphatase; ALT:alanine aminotransferase; Diabetes-1:diabetes mellitus type 1. including LADA; Diabates-2: diabetes mellitus type 2 treated with oral medications; Diabetes-3: diabetes mellitus type 2 treated with insulin; Diabtes-4: prediabetes; COPD: chronic obstructive pulmonary disease; TIA: transient ischemic attack

Table S21. Model for TBIL and ALT adjusted for comorbidities and potential risk factors, n=401.

	**ICU admission**							**Mechanical ventilation**							**Fatal outcome**						
	**OR**	**Cl 95%**		**p-value**	**p-value for model**	**NG**	**LR p-value**	**OR**	**Cl 95%**		**p-value**	**p-value for model**	**NG**	**LR p-value**	**OR**	**Cl 95%**		**p-value**	**p-value for model**	**NG**	**LR p-value**
		**2.5%**	**97.5%**						**2.5%**	**97.5%**						**2.5%**	**97.5%**				
TBIL	0.950	0.684	1.260	0.731	0.001	0.238	<0.0001	0.968	0.699	1.280	0.825	0.0003	0.242	<0.0001	1.556	1.155	2.190	0.007	<0.0001	0.183	<0.0001
ALT	1.000	0.998	1.000	0.523				1.000	0.999	1.000	0.449				1.001	1.000	1.000	0.286			
Hypertension	1.200	0.726	1.980	0.483				1.480	0.891	2.480	0.132				1.751	1.040	2.980	0.037			
Diabetes -1	0.240	0.012	1.520	0.201				0.249	0.013	1.590	0.215				0.712	0.130	3.070	0.666			
Diabetes -2	1.090	0.567	2.060	0.803				1.110	0.573	2.110	0.763				1.480	0.802	2.710	0.205			
Diabetes -3	1.180	0.437	3.090	0.733				1.340	0.487	3.560	0.564				0.912	0.358	2.220	0.841			
Diabetes - 4	1.370	0.321	5.670	0.663				1.380	0.320	5.770	0.659				2.865	0.710	11.670	0.131			
Asthma	1.410	0.408	4.710	0.573				1.030	0.276	3.460	0.966				0.860	0.178	3.030	0.830			
COPD	<0.001	<0.001	>1000	0.992				<0.001	<0.001	>1000	0.992				0.847	0.187	3.270	0.816			
Dementia	<0.001	<0.001	>1000	0.987				<0.001	<0.001	>1000	0.986				3.178	1.296	8.120	0.013			
Stroke/TIA in patient history	0.661	0.254	1.580	0.370				0.740	0.284	1.790	0.517				1.026	0.464	2.210	0.949			

Chronic kiedney disease	0.334	0.160	0.660	0.002				0.323	0.152	0.640	0.002				0.799	0.434	1.440	0.463			
Active smoker	0.534	0.131	1.840	0.341				0.397	0.082	1.470	0.198				0.922	0.293	2.680	0.884			
Former smoker	0.413	0.106	1.320	0.161				0.321	0.069	1.100	0.098				0.739	0.233	2.140	0.589			
Myocardial infraction in patient history	1.670	0.776	3.600	0.187				1.570	0.712	3.430	0.261				2.786	1.422	5.540	0.003			
Heart failure	0.834	0.391	1.730	0.630				0.607	0.274	1.290	0.205				1.253	0.658	2.370	0.488			
Leukemia	2.930	0.688	13.190	0.143				1.100	0.205	4.840	0.900				4.976	1.215	24.950	0.031			
Solid malignant disease without metastases	0.783	0.269	2.120	0.638				0.658	0.214	1.810	0.436				1.457	0.561	3.690	0.428			
Solid malignant disease with metastases	<0.001	<0.001	>1000	0.993				<0.001	<0.001	>1000	0.993				0.095	0.004	0.690	0.053			
Lymphoma	<0.001	<0.001	>1000	0.992				<0.001	<0.001	>1000	0.992				0.643	0.082	3.250	0.624			
Lenght of hospital stay	1.020	1.010	1.030	0.004				1.020	1.010	1.030	0.005				0.993	0.979	1.010	0.288			

OR: Odds Ratio; 95% CI: Confidence Interval; NG: Nagelkerke pseudo R2; LR: Likelihood Ratio; ICU: intensive care unit; TBIL: total bilirubin; ALT:alanine aminotransferase; Diabetes-1:diabetes mellitus type 1. including LADA; Diabates-2: diabetes mellitus type 2 treated with oral medications; Diabetes-3: diabetes mellitus type 2 treated with insulin; Diabtes-4: prediabetes; COPD: chronic obstructive pulmonary disease; TIA: transient ischemic attack

Table S22. Model for TBIL and ALT adjusted for comorbidities and potential risk factors, n=401.

	ICU admission							Mechanical ventilation							Fatal outcome						
	OR	Cl 95%		p-value	p-value for model	NG	LR p-value	OR	Cl 95%		p-value	p-value for model	NG	LR p-value	OR	Cl 95%		p-value	p-value for model	NG	LR p-value
		2.5%	97.5%						2.5%	97.5%						2.5%	97.5%				
TBIL	1.094	0.732	1.620	0.651	0.002	0.240	<0.0001	1.112	0.744	1.650	0.595	0.001	0.243	<0.0001	1.580	1.128	2.280	0.010	<0.0001	0.177	0.0001
ALP	0.998	0.995	1.000	0.352				0.999	0.995	1.000	0.384				1.000	0.997	1.000	0.875			
Hypertension	1.180	0.718	1.950	0.515				1.454	0.877	2.430	0.149				1.710	1.020	2.900	0.044			
Diabetes -1	0.275	0.014	1.820	0.253				0.285	0.014	1.910	0.269				0.701	0.125	3.160	0.660			
Diabetes -2	1.103	0.575	2.090	0.765				1.123	0.581	2.150	0.727				1.478	0.801	2.700	0.207			
Diabetes -3	1.209	0.448	3.150	0.700				1.369	0.501	3.640	0.531				0.936	0.368	2.270	0.887			
Diabetes - 4	1.361	0.319	5.640	0.667				1.366	0.318	5.730	0.665				2.831	0.702	11.520	0.135			
Asthma	1.670	0.496	5.540	0.396				1.233	0.344	4.110	0.735				0.962	0.208	3.310	0.955			
COPD	<0.001	<0.001	>1000	0.992				<0.001	<0.001	>1000	0.992				0.812	0.179	3.140	0.771			
Dementia	<0.001	<0.001	>1000	0.988				<0.001	<0.001	>1000	0.988				3.443	1.430	8.680	0.007			
Stroke/TIA in patient history	0.635	0.244	1.520	0.325				0.710	0.272	1.710	0.461				1.002	0.453	2.160	0.996			

Chronic kiedney disease	0.339	0.163	0.670	0.003	0.328	0.155	0.650	0.002	0.786	0.426	1.420	0.430
Active smoker	0.528	0.130	1.820	0.333	0.394	0.081	1.460	0.195	0.943	0.303	2.720	0.915
Former smoker	0.408	0.104	1.300	0.155	0.317	0.068	1.090	0.094	0.735	0.233	2.130	0.582
Myocardial infraction in patient history	1.653	0.767	3.570	0.198	1.538	0.699	3.370	0.281	2.734	1.396	5.440	0.004
Heart failure	0.817	0.382	1.700	0.593	0.597	0.269	1.270	0.190	1.251	0.657	2.360	0.492
Leukemia	3.027	0.705	13.700	0.133	1.118	0.205	4.950	0.887	4.871	1.190	24.420	0.033
Solid malignant disease without metastases	0.820	0.280	2.240	0.705	0.689	0.223	1.910	0.491	1.418	0.546	3.590	0.463
Solid malignant disease with metastases	<0.001	<0.001	>1000	0.993	<0.001	<0.001	>1000	0.993	0.099	0.004	0.710	0.055
Lymphoma	<0.001	<0.001	>1000	0.992	<0.001	<0.001	>1000	0.992	0.613	0.077	3.120	0.589
Lenght of hospital stay	1.021	1.007	1.040	0.003	1.021	1.007	1.040	0.004	0.992	0.978	1.010	0.257

OR: Odds Ratio; 95% CI: Confidence Interval; NG: Nagelkerke pseudo R2; LR: Likelihood Ratio; ICU: intensive care unit; TBIL: total bilirubin; ALP:alkaline phosphatase; Diabetes-1:diabetes mellitus type 1. including LADA; Diabates-2: diabetes mellitus type 2 treated with oral medications; Diabetes-3: diabetes mellitus type 2 treated with insulin; Diabtes-4: prediabetes; COPD: chronic obstructive pulmonary disease; TIA: transient ischemic attack
